# Dual Modulation of Canonical and Non-canonical TGF-β/ROS/Erk1/2 Pathways: Synergistic Activation of Nrf-2 and Antioxidant Enzymes (SOD1, GPx, HO-1) by Quercetin Loaded in Solid Lipid Nanoparticles and Curcumin in Atherosclerosis Therapy

**DOI:** 10.5812/ijpr-151428

**Published:** 2024-12-13

**Authors:** Masoumeh Shamsi, Ghorban Mohammadzadeh, Mahdi Hatami, Mohammadreza Roshanazadeh, Mojgan Noor-Behbahani, Mojtaba Rashidi

**Affiliations:** 1 Department of Clinical Biochemistry, Hyperlipidemia Research Center, School of Medicine, Ahvaz Jundishapur University of Medical Sciences, Ahvaz, Iran; 2 Department of Clinical Biochemistry, Faculty of Medicine, Ahvaz Jundishapur University of Medical Sciences, Ahvaz, Iran

**Keywords:** Atherosclerosis, Vascular Smooth Muscle Cells (VSMCs), Transforming Growth Factor–β, Nano-Quercetin, Curcumin, Combination Therapy

## Abstract

**Background:**

Atherosclerosis remains the leading cause of mortality worldwide, highlighting the urgent need for innovative treatments targeting chronic inflammation. Recent research indicates that quercetin (QCT) and curcumin, two naturally occurring compounds, have potential therapeutic benefits in cardiovascular diseases.

**Objectives:**

This study focuses on the novel synthesis of nano-quercetin (N-QCT) encapsulated in solid lipid nanoparticles (SLNs) and investigates the synergistic cardioprotective effects of N-QCT and curcumin on human vascular smooth muscle cells (VSMCs). The underlying molecular mechanisms, particularly the involvement of the TGF-β signaling pathway in VSMCs, are explored.

**Methods:**

The VSMCs, including TGF-β-stimulated VSMCs, were treated with N-QCT, curcumin, or a combination of both. The MTT assay was performed to evaluate the cytotoxic effects of these treatments. The cytotoxicity of various concentrations of curcumin and QCT was used to calculate the Combination Index (CI), with CI analysis quantifying synergy or antagonism. Furthermore, following TGF-β stimulation, antioxidant enzyme activity, nuclear transcription factor erythroid 2-related factor (Nrf2) mRNA expression, reactive oxygen species (ROS) production, NADPH oxidases (NOX) expression, and extracellular signal-regulated kinase (Erk)1/2 phosphorylation were measured in the treated VSMCs.

**Results:**

The N-QCT and curcumin significantly influenced Nrf2 mRNA expression and upregulated downstream antioxidant enzymes, including HO-1, GPx, and SOD1. The combination treatment further enhanced Nrf2 protein expression and modulated Erk1/2 phosphorylation. Notably, the synergistic effect of the combination produced pronounced cardioprotective outcomes, characterized by reduced ROS production and decreased phosphorylation of Erk1/2 via the TGF-β/NOX/Erk1/2 and ROS/Nrf2 signaling pathways.

**Conclusions:**

The findings demonstrate that the combination of QCT encapsulated in SLNs and curcumin synergistically reduces oxidative stress and inflammation in TGF-β-stimulated VSMCs. This effect is achieved through the inhibition of ROS/Erk1/2 signaling and the activation of Nrf2 and antioxidant enzymes. These natural compounds, when used together, represent a promising therapeutic approach for mitigating the inflammatory processes associated with atherosclerosis.

## 1. Background

Atherosclerosis, the leading cardiovascular disease worldwide, accounts for 32% of global deaths. It increases the risk of heart attack and stroke due to fat deposition and inflammation in large arteries. Over time, lipids, inflammatory cells, and vascular smooth muscle cells (VSMCs) accumulate, altering the endothelial cell layer of the inner artery wall and releasing cytokines and necrotic cell debris (1). Cytokines such as TGF-β play a crucial role in the development of advanced atherosclerotic lesions by promoting VSMC proliferation (2, 3). 

TGF-β signaling involves multiple pathways, including the Smad pathway, non-Smad pathways, and sequential pathways like mitogen-activated protein kinase (MAPK) and extracellular signal-regulated kinase (Erk). The non-Smad pathway induces NADPH oxidases (NOXs) and reduces mitochondrial activity, leading to increased reactive oxygen species (ROS) levels (4, 5). TGF-β activates signaling by binding to the intracellular kinase domains of transmembrane receptor types I and II. Type II receptor kinases phosphorylate and activate type I receptors (6, 7). 

Mediators of the TGF-β signaling pathway can positively or negatively influence its biological outcomes (8). Furthermore, while TGF-β stabilizes atherosclerotic plaques (9), ROS plays a central role in the formation of atherosclerosis (10). 

NADPH oxidases, an intermediate product, is generated through the high expression or activity of NOX enzymes in the Smad pathway. This process results in ROS production, leading to protein damage, cell membrane damage, and DNA damage, all of which contribute to the development of atherosclerosis (11). Before the discovery of Smads, TGF-β was shown to cause rapid activation of Ras in normal epithelial cells, suggesting that Erk MAPKs were involved in TGF-β signaling (12). 

TGF-β promotes Erk1/2 activation and phosphorylation, which serves as a key signal transducer for cell growth and the progression of atherosclerosis (13). However, it can suppress the antioxidant network, including enzymes such as superoxide dismutase, glutathione peroxidase, and catalase (14). The inducible phase II detoxification response is mediated by the nuclear transcription factor erythroid 2-related factor (Nrf2), which activates enzymes like heme oxygenase-1 and glutamate-cysteine ligase, providing protection against oxidative stress (15, 16). 

Thus, drugs that inhibit the intracellular NOX signaling pathway and modulate the Erk/Nrf2/ARE axis may effectively prevent the progression of atherosclerosis (17, 18). 

Curcumin, a compound found in turmeric, is a free radical inhibitor and the primary pigment in the yellow Indian spice herb. In addition to its anti-inflammatory, wound-healing, and antioxidant properties, curcumin can directly influence gene expression and signaling pathways (19). Recent research further supports its ability to modulate these processes (20). 

Quercetin, a flavonoid present in fruits and vegetables such as apples, strawberries, grapes, broccoli, black tea, and onions, is a well-studied compound with biological properties that play a protective role in the cardiovascular system (21). Studies indicate that dietary flavonoids, including quercetin (QCT), can reduce the risk of cardiovascular disease (22). Quercetin has been shown to exert various biological effects, including improving ischemia, inhibiting smooth muscle relaxation, reducing cholesterol aggregation, and preventing LDL oxidation. Additionally, it inhibits cell migration and proliferation in cancer cells, reduces the expression of heat shock proteins, prevents reperfusion injury, and promotes overall health (23). 

Despite its anti-inflammatory, anti-atherogenic, and anti-hypertensive properties, which make it valuable in treating cardiovascular disease, QCT's lipophilic nature and low water solubility limit its efficacy and clinical application (24). 

Optimizing a drug delivery system to regulate QCT's effects is essential, with solid lipid nanoparticles (SLNs) gaining attention due to their extensive applications in biology and medicine (25). Nanoparticles used for drug delivery must exhibit biocompatibility, drug tolerance, biodegradability, controlled release, favorable mechanical properties, and a straightforward manufacturing process (26). The SLNs are particularly versatile, avoiding the use of organic solvents, accommodating both lipophilic and hydrophilic drugs, and being suitable for large-scale production and effective sterilization. Additionally, biocompatible carriers enhance both drug stability and physical stability (27). 

## 2. Objectives 

This study explored the connection between TGF-β/TGFBR signaling and NOX/ROS-mediated NOX production, which may contribute to increased vascular inflammation. It also examines the effects of nano-quercetin (N-QCT) and curcumin on p-ERK1/2 and p-Nrf2 antioxidant protein expression in TGF-β-stimulated VSMCs. The study's objective is to elucidate the molecular mechanisms underlying QCT's role and its function in the development of atherosclerosis. 

## 3. Methods

### 3.1. Substances and Agents

Fetal bovine serum (FBS) and Dulbecco's Modified Eagle's Medium (DMEM) were purchased from GIBCO (Invitrogen, Carlsbad, CA, USA). Trypsin-EDTA and penicillin/streptomycin antibiotics were obtained from Ideazist (Iran). Quercetin, curcumin, oleic acid, lecithin, and polyvinyl alcohol (PVA) were procured from Sigma-Aldrich (St. Louis, MO, USA). For the protein assay, the bicinchoninic acid (BCA) kit was acquired from Parstous (Mashhad, Iran). Recombinant TGF-β and a range of antibodies, including HRP Anti-Rabbit IgG peroxidase, Anti-phospho-Nrf2 (Ser40), Anti-phospho-ERK1/2 (Thr202/Tyr204), and GAPDH, were sourced from Cell Signaling Technology (Beverly, MA, USA). 

### 3.2. Preparation of Curcumin Stock Solution 

To prepare a curcumin stock solution in DMSO at an initial concentration of 50 mM, the following procedure was used: Curcumin was first dissolved at a concentration of 5 mM in DMSO. The mixture was maintained at 20°C and stored in the dark to ensure stability. The curcumin stock solution was then diluted with culture media to achieve final working concentrations of 10, 25, 50, 100, and 150 μM for cell treatments, respectively. 

### 3.3. Preparation of Nano-Quercetin

To prepare N-QCT, a mixture of 100 mg of QCT and 4.75 grams of Compritol 888 ATO (a solid lipid, Glyceryl Dibehenate, Grabefossé) was heated to 75°C. Separately, deionized water was combined with 0.25 grams of oleic acid (as a liquid lipid) and 0.5 grams of lecithin (as an emulsifier), heated to 80°C, and stirred for 5 minutes. The mixture was then homogenized at 10000 rpm using an Elmasonic S60H homogenizer (Heidolph Schüttler, Germany). 

The resulting solution and the original mixture were further sonicated using an ultrasonicator (Global Industrial, USA). To create a nanoemulsion, 4 mL of a 1% PVA solution (as a surfactant) was added to the mixture at 3°C. The mixture was again homogenized at 10000 rpm using a Heidolph homogenizer. Subsequently, the suspension was centrifuged twice for 20 minutes at 25,000 RCF and 5°C. The prepared N-QCTs were stored in sealed containers in a refrigerator until needed. 

### 3.4. Transmission Electron Microscopy 

Transmission electron microscopy (TEM) was employed to analyze the SLN structure. A drop of the SLN suspension was placed onto a carbon-coated copper grid to form a thin liquid layer. The samples were air-dried for five minutes at room temperature before being analyzed by TEM. 

### 3.5. Measuring Infrared with the Fourier Transform 

FTIR spectrum analysis was performed to confirm the interactions in the N-QCT preparation using a VERTEX 70 V spectrometer (Bruker, USA). The chemical interaction between SLNs and QCT was validated based on the obtained spectra. Samples were pressed into pellets with KBr under a pressure of 200 kg/cm^2^. The FTIR spectra of QCT, N-QCT, and blank SLNs were recorded at a resolution of 1 cm^-1^, covering a range from 400 to 4000 cm^-1^.

### 3.6. Zeta Potential and Particle Dimensions of Nano-Quercetin

The N-QCTs were characterized for average particle size, zeta potential, and Polydispersity Index (PDI) using a Nanosizer and Zetasizer (Malvern, England). The morphology was examined with a transmission electron microscope (ZEISS LEO 906 E, Germany). For sample preparation, a thin layer of SLN suspension was deposited onto a carbon-coated copper grid. The samples were air-dried at room temperature for five minutes after removing excess liquid with paper filters. The size and shape of the SLNs on these dried samples were then measured and observed using the transmission electron microscope. 

### 3.7. Encapsulation Efficiency In Vitro 

The N-QCT suspension was centrifuged at 25000 rpm for 25 minutes, and the quantity of QCT in the supernatant was measured using a UV spectrophotometer (Pharmacia Biotech, USA, Ultraspec 3000) at a wavelength of 256 nm. The encapsulation efficiency (EE) was calculated using the following formula: 

EE (%) = 100 (Initial drug concentration - Not included drug concentration)/Initial drug concentration

To calculate the drug loading (DL), N-QCT was first dissolved in methanol, and the amount of QCT in the solution was then measured with the spectrophotometer at 256 nm. Drug loading (DL) was calculated using the formula:


$$DL\;\left(\%\right)=100\;\times \frac{\left(W_{i}-W_{f}\right)}{W_{l}}$$

Where, W_i_ represents the weight of the initial drug, W_f_ represents the weight of the drug remaining after the free drug is removed from the supernatant, and W_l_ is the weight of the lipid used to produce the nanoparticles. 

### 3.8. Drug Release In Vitro 

The release of QCT from N-QCT was assessed using the dialysis bag method. A phosphate buffer solution (pH 7.4) with a molecular weight cutoff of 12000 Da (Sigma-Aldrich, USA) was used as the receptor phase, maintained at 37°C. Samples were collected at regular intervals and analyzed spectrophotometrically at 256 nm. 

### 3.9. Experimental Design for Vascular Smooth Muscle Cells Culture and Treatment 

Human VSMCs were purchased from the Pasteur Institute in Tehran, Iran. The VSMC culture was supplemented with 100 U/mL streptomycin, 100 mg/mL penicillin, and 10% FBS. The VSMCs were maintained at 37°C in a humidified atmosphere with 5% CO_2_. After seeding, the cells were divided into several groups: (1) Control group (media only), (2) N-QCT-only group, (3) curcumin-only group, (4) TGF-β-treated group, (5) N-QCT in the TGF-β-induced VSMC group, (6) curcumin in the TGF-β-induced VSMC group, and (7) combination of N-QCT and curcumin in the TGF-β-induced VSMC group. In this experiment, the effects of N-QCT and curcumin on VSMCs were also evaluated in the absence of TGF-β stimulation, serving as control groups (groups 2 and 3). 

### 3.10. In Vitro Cytotoxicity Assay 

The MTT assay was used to assess the viability of VSMCs. The VSMCs were seeded in 96-well plates at a density of 5 × 10³ cells per well and incubated overnight at 37°C in a 5% CO₂ atmosphere to facilitate cell adhesion. The cells were then cultured for an additional 24 and 48 hours with various concentrations of N-QCT (10, 25, 50, 100, and 150 μM) and curcumin (10, 25, 50, 100, and 150 μM). After treatment, the media was aspirated, and 0.5 mg/mL MTT solution was added to the cells. The plates were incubated in the dark for four hours. Following incubation, the formazan crystals formed were dissolved by adding 150 μL of dimethyl sulfoxide (DMSO) (Merck, Darmstadt, Germany) to each well. The plates were shaken for fifteen minutes, and the optical density (OD) of each well was measured at 570 nm using a BioTek ELx800 microplate reader (Winooski, Vermont, USA). The percentage of cell viability was calculated by comparing the absorbance of treated cells with that of untreated control cells. Data analysis and determination of the half-maximal inhibitory concentration (IC₅₀) were performed using GraphPad Prism version 8.0 (La Jolla, CA). This software was used to process the experimental data and calculate the IC₅₀ values for curcumin and N-QCT treatments.

### 3.11. Investigation of Combination Index 

CompuSyn (Chou and Martin, 2005; Compusyn Inc., USA) was used to calculate the CI and evaluate the cooperative relationship between curcumin and N-QCT. Combination Index values > 1 and < 1 indicate antagonism and synergy, respectively, while CI = 1 indicates additive effects. The Chou method was applied for CI analysis (28, 29). 

CI = D_1_/Dx_1_ + D_2_/Dx_2_

The IC₅₀ values D_1_ and D_2_ of each combination drug are determined by calculating the mole fraction of the IC₅₀ value; the IC₅₀ values of a single drug are denoted as Dx_1_ and Dx_2_ (28). Furthermore, the CI has four classifications based on values: High synergism (CI between 0.1 and 0.3), moderate synergism (CI between 0.3 and 0.7), mild synergism (CI between 0.7 and 0.85), and no synergism (CI between 0.85 and 1.0). 

### 3.12. Detection of Reactive Oxygen Species Production 

The ROS assay was performed using the 2′,7′-dichlorodihydrofluorescein (H₂DCF) probe and a fluorimetric technique to measure the production of 7′-dichlorofluorescein (DCF), indicative of H₂O₂ levels produced by the cells. The fluorescence emitted by DCF was measured at 500 and 600 nm. Human VSMCs were cultured in 12-well plates and treated with TGF-β (2 ng/mL), N-QCT, curcumin, and a combination of both. After a 24-hour incubation, H₂DCF solution (25 μM) was added, and fluorescence was measured using a spectrofluorometer (Cary, Australia) with excitation at 485 nm and emission at 535 nm.

### 3.13. Quantitative Real-Time Polymerase Chain Reaction

The real-time PCR technique (Amplicon, Denmark) was used to determine the expression of genes encoding NOX1, NOX2, NOX4, Nrf2, and antioxidant enzymes. After collecting the cells, total RNA was extracted using an RNA isolation kit (Yectatajhizazma) following the manufacturer's instructions. RNA concentration and purity were determined by measuring absorbance at A260 nm/A280 nm using a Nanodrop 2000 (Thermo Fisher Scientific). cDNA synthesis was then performed using a cDNA synthesis kit (Yectatajhizazma, Iran). The primers listed in Table 1 were used for gene expression, and real-time PCR amplification was performed using a Biosystem (initial denaturation at 95°C for 15 minutes, followed by 40 cycles of 15 seconds at 95°C and 1 minute at 60°C for denaturation and annealing, respectively). GAPDH primers were used as the housekeeping gene (F: 5'-CATCACTGCCACCCAGAAGACTG-3', R: 5'-ATGCCAGTGAGCTTCCCGTTCAG-3'). The comparative delta-delta cycle threshold (ΔΔCt) method was employed to assess the fold variation in mRNA expression from the qRT-PCR tests.

**Table 1.** Primer Sequences

Target Gene	Primer Sequence 5 to 3	Gene Bank Accession Number	Length, bp
**NOX1**	F-GGTTTTACCGCTCCCAGCAGAA	NM_007052	101
	R-CTTCCATGCTGAAGCCACGCTT		
**NOX2**	F-CTCTGAACTTGGAGACAGGCAAA	NM_000397	131
	R-CACAGCGTGATGACAACTCCAG		
**NOX4**	F-GCCAGAGTATCACTACCTCCAC	NM_016931	112
	R-CTCGGAGGTAAGCCAAGAGTGT		
**Nrf2**	F-AGGTTGGAGCTGTTGATCTGT	NM_001145412.3	138
	R-AATCCATGTCCCTTGACAGCA		
**SOD-1**	F-GGCAATGTGACTGCTGACAA	NM_000454.4	116
	R-GCTTTTTCATGGACCACCAGT		
**HO-1**	F-CCAGGCAGAGAATGCTGAGTTC	NM_002133	143
	R-AAGACTGGGCTCTCCTTGTTGC		
**GPx**	F-GTGCTCGGCTTCCCGTGCAAC	NM_000581	122
	R-CTCGAAGAGCATGAAGTTGGGC		

Abbreviations: NOX, NADPH oxidases; Nrf2, nuclear transcription factor erythroid 2-related factor.

### 3.14. Western Blot Technique

The study involved VSMC cells exposed to various treatments for 48 hours, including N-QCT and curcumin. The cells were then lysed using RIPA lysis buffer containing a protease inhibitor to prevent protein degradation. Proteins were separated using SDS-PAGE and transferred to a polyvinylidene fluoride (PVDF) membrane for 120 minutes. The membrane was blocked for 75 minutes with Tris-buffered saline containing 0.1% Tween 20 (TBST) and 5% nonfat dry milk. Specific primary antibodies against Erk1/2 (1:1000; sc-514302), Nrf2 (1:1000; #12721), and GAPDH (1:1000; sc-5174) (Santa Cruz Biotechnology, USA) were incubated on the membrane overnight at 4°C. The membrane was then exposed to a goat anti-rabbit IgG secondary antibody conjugated with peroxidase for 1 hour at room temperature. Protein bands were detected using the Enhanced Chemiluminescence (ECL) kit from Abcam, which facilitates protein detection via chemiluminescence. The intensity of the protein bands was quantified using the ImageJ program developed by the National Institutes of Health (NIH), allowing for the analysis of protein expression levels.

### 3.15. Statistical Analysis 

The results of the three independent experiments were presented as the mean ± standard error of the mean (SEM). Data were managed and analyzed using GraphPad Prism. ANOVA was used to examine the data, and P < 0.05 and P < 0.01 were considered statistically significant. A post hoc least significant difference (LSD) analysis was then performed.

## 4. Results

### 4.1. Characterization of Nano-Quercetin

The formulation of QU-SLN is provided in Appendix 4 in Supplementary File. Based on dynamic light scattering (DLS) measurements, the average nanoparticle size was found to be 154 ± 22.5 nm (Appendix 1 in Supplementary File). The long-term stability of the nanoparticles was ensured by their negative zeta potential of approximately -27.7 mV, which prevents aggregation (Appendix 2 in Supplementary File). The Polydispersity Index (PDI) of N-QCT was 0.50 ± 0.04, indicating that the nanoparticles in the sample were uniformly distributed.

### 4.2. Transmission Electron Microscopy Image 

**Figure 1.**
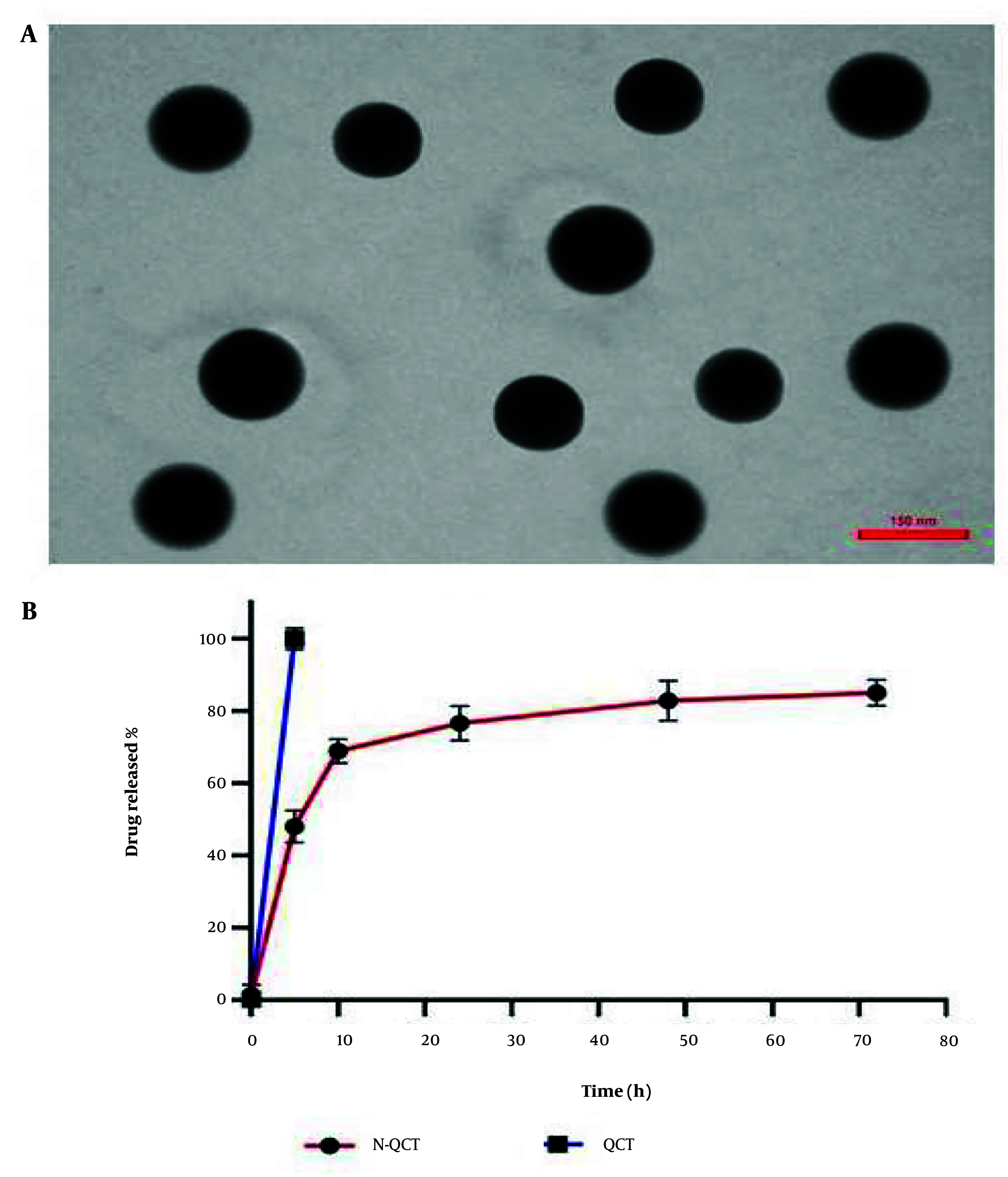
A, a micrograph of nano-quercetin (N-QCT) produced by TEM; B, cumulative percentage of N-QCT released in the assay.

Figure 1A shows spherical nanoparticles with smooth surfaces, as observed in the TEM images. Notably, the average particle size was below the estimated 156 nm. This powerful imaging technique allowed for a detailed examination of the structure and properties of N-QCT particles at the nanoscale level.

### 4.3. FTIR Analysis 

The FTIR test results are shown in Appendix 3 in Supplementary File. These results reveal that QCT has characteristic peaks at O-H stretching (3850 - 3200 cm^-1^), C = O stretching (1633 cm^-1^), C-C stretching (1659 cm^-1^), and C-H stretching (1663, 1379 cm^-1^). Additional peaks include C-O stretching in the ring structure (1263 cm^-1^) and C-O stretching in the ring structure (1109 - 1056 cm^-1^). The absence of any shifts in the functional group peaks of N-QCT confirms its proper structure in conjunction with the other compounds used in the preparation.

### 4.4. Drug Release In Vitro 

The in vitro release profile demonstrated an initial rapid release within 0.5 to 6 hours, followed by a gradual release of QCT from N-QCT over 48 hours. This suggests that N-QCT may provide sustained QCT release during treatment. The release patterns were consistent across different formulations. The cumulative percentage of QCT released from the N-QCT formulation is shown in Figure 1B. Unlike free QCT, which has low solubility in aqueous media (Figure 1B), N-QCT was fully dispersed in water without forming aggregates. 

**Figure 2.**
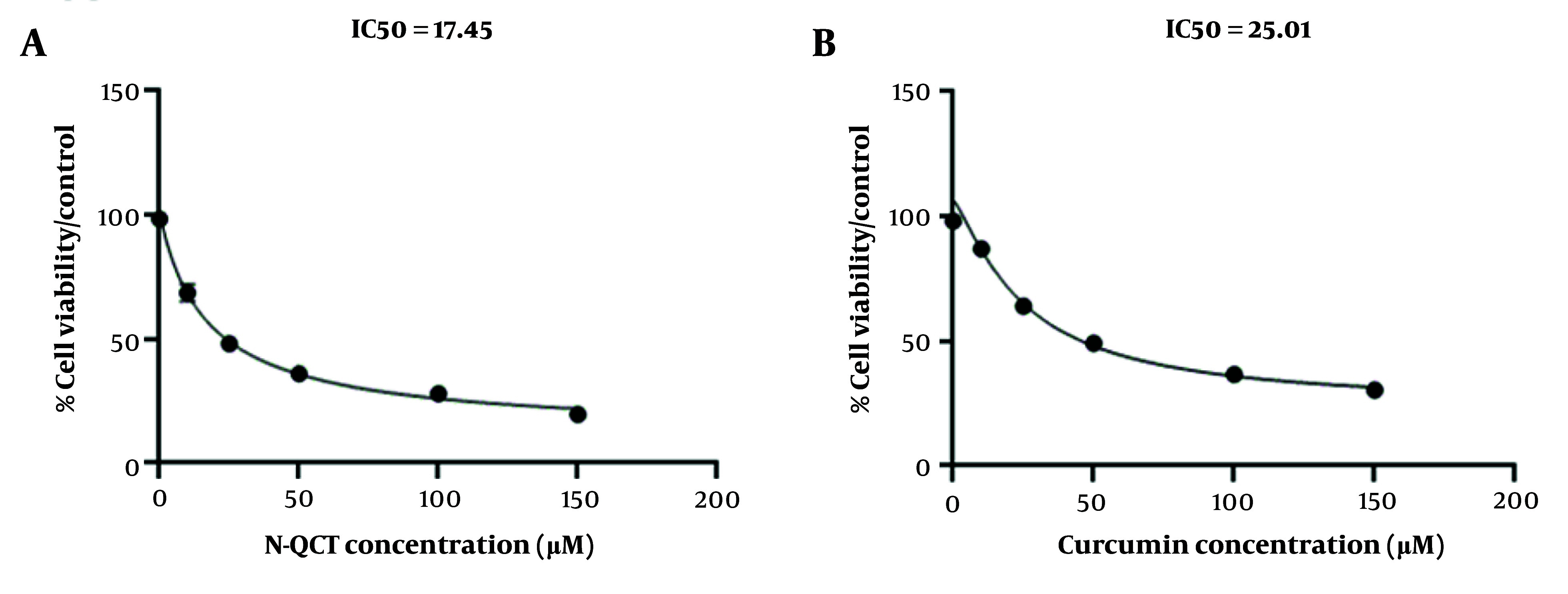
The IC_50 _values of nano-quercetin (N-QCT) and curcumin on vascular smooth muscle cells (VSMCs) were determined using the MTT assay. A, cells were exposed to varying concentrations of N-QCT (0 - 150 μM/mL) for 48 hours to assess its dose-dependent cytotoxicity; B, similarly, VSMCs were treated with different concentrations of curcumin (0 - 150 μM/mL) for 48 hours.

### 4.5. Cell Viability 

The IC_50_ values for N-QCT and curcumin were determined to evaluate their inhibitory effects on VSMC cell growth after 48 hours of exposure, as shown in Figure 2A and B. The IC_50_ value for N-QCT was significantly lower than that of curcumin at different time points. The IC_50_ value for N-QCT against VSMC cells was 17.45 ± 2.09 μM, while the IC_50_ value for curcumin against VSMC cells was 25.01 ± 2.00 μM (Figure 2A and B). Based on data showing cell viability with varying concentrations of N-QCT and curcumin (Appendix 5 in Supplementary File), the CI was calculated using the formula: 

CI [N-QCT + curcumin] = 17.45/50 (μM) + 25.01/50 (μM) = 0.866

This result suggests that the combination of N-QCT and curcumin exhibits a mild to moderate synergistic effect. To determine the optimal concentrations for combining N-QCT and curcumin, the CI results can be used, with concentrations near the IC_50_ values recommended for more effective combinations.

### 4.6. TGF-β Induces Reactive Oxygen Species Production in Human Vascular Smooth Muscle Cells

**Figure 3.**
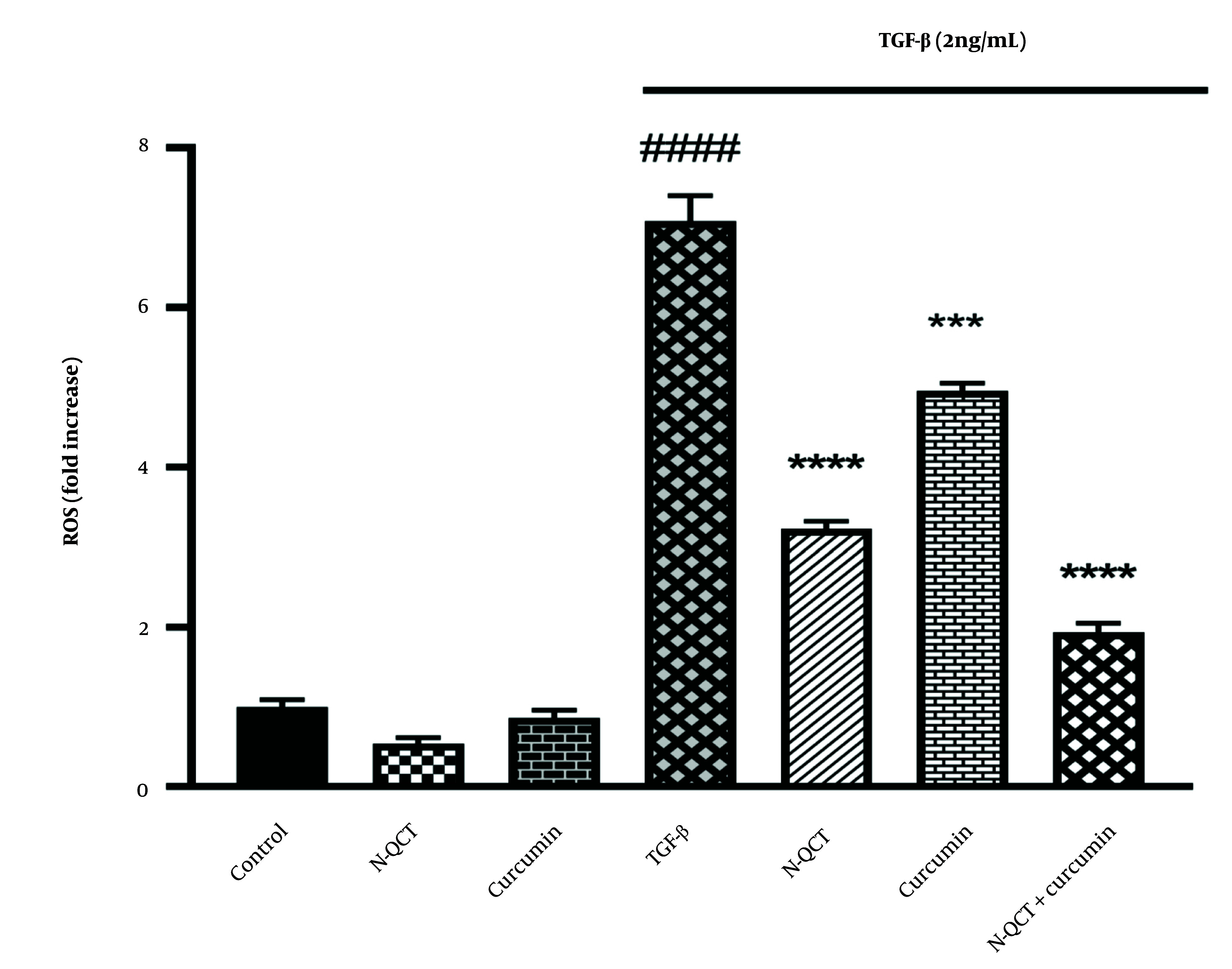
Reactive oxygen species (ROS) production in vascular smooth muscle cells (VSMCs) was assessed after treatment with nano-quercetin (N-QCT), curcumin, or their combination for 24 hours, with or without TGF-β (2 ng/mL). Statistical significance is indicated as follows: #### P < 0.0001 for comparisons between the TGF-β-stimulated group and control cells; and *** P < 0.001, **** P < 0.0001 for comparisons between other treatment groups and the TGF-β-treated cells (mean ± SD). The results were analyzed after three repetitions.

There was a significant increase in ROS levels in VSMC cells treated with TGF-β (2 ng/mL) compared to the control group (P < 0.0001). In contrast, the ROS levels in the groups treated with either N-QCT or curcumin alone were comparable to those in the untreated group. As shown in Figure 3, the group treated with both N-QCT and curcumin in TGF-β-stimulated VSMCs exhibited a significant reduction in ROS levels compared to the TGF-β (2 ng/mL) group (P < 0.001). These results demonstrate that TGF-β significantly enhances the production of ROS, highlighting its role in amplifying oxidative stress. This increase in ROS production underscores the impact of TGF-β on intracellular pathways in VSMCs, suggesting that TGF-β-induced oxidative stress may influence various cellular signaling mechanisms and contribute to pathological changes within these cells.

### 4.7. mRNA Expression of NADPH Oxidases in TGF-β-Stimulated Vascular Smooth Muscle Cells

**Figure 4.**
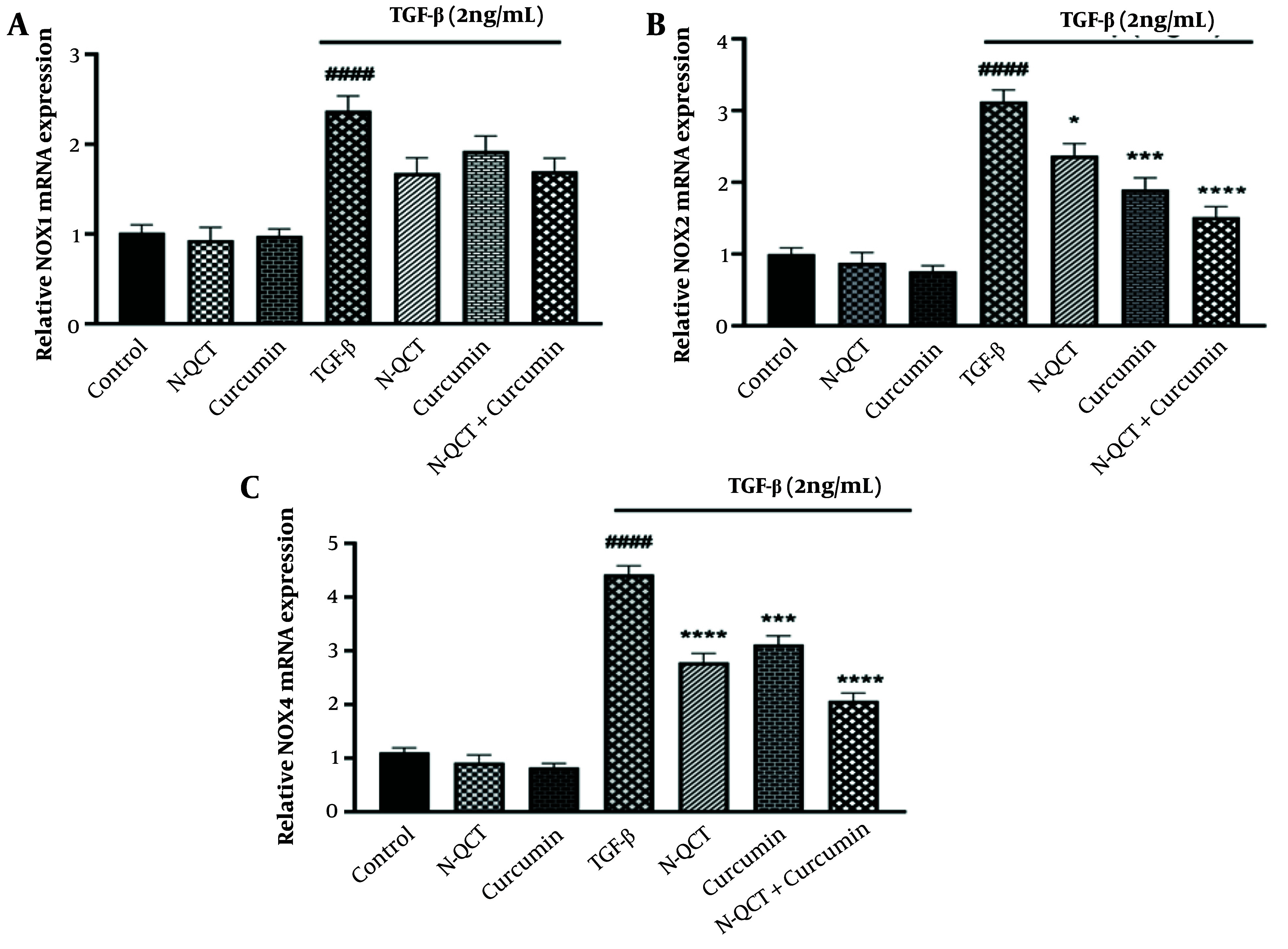
mRNA expression levels of NADPH oxidases (NOX) genes [NOX1, NOX2, and NOX4, labeled as (A), (B), and (C), respectively] in vascular smooth muscle cells (VSMCs), with and without TGF-β (2 ng/mL), following treatment with nano-quercetin (N-QCT), curcumin, or a combination of both. Statistical significance is indicated as follows: # (#### P < 0.0001) for comparisons between the TGF-β-stimulated group and control cells, and * P < 0.05, *** P < 0.001, **** P < 0.0001 for comparisons between other treatment groups and the TGF-β-treated cells (mean ± SD). The results were analyzed after three repetitions.

 The VSMCs treated with TGF-β showed significantly increased expression of the genes NOX1, NOX2, and NOX4 compared to the untreated group (P < 0.0001). In TGF-β-stimulated VSMCs exposed to N-QCT and curcumin alone, the expression of NOX1 showed a slight decrease, while NOX2 and NOX4 exhibited a significant decrease compared to the TGF-β (2 ng/mL) treated group. After combination treatment, there was a significant reduction in the expression of NOX1, NOX2, and NOX4 genes compared to the TGF-β-stimulated VSMC group (Figure 4A - C).

### 4.8. mRNA Expression of Antioxidant Enzymes in the Reactive Oxygen Species-Dependent Signaling Pathway in TGF-β-Stimulated Vascular Smooth Muscle Cells

**Figure 5.**
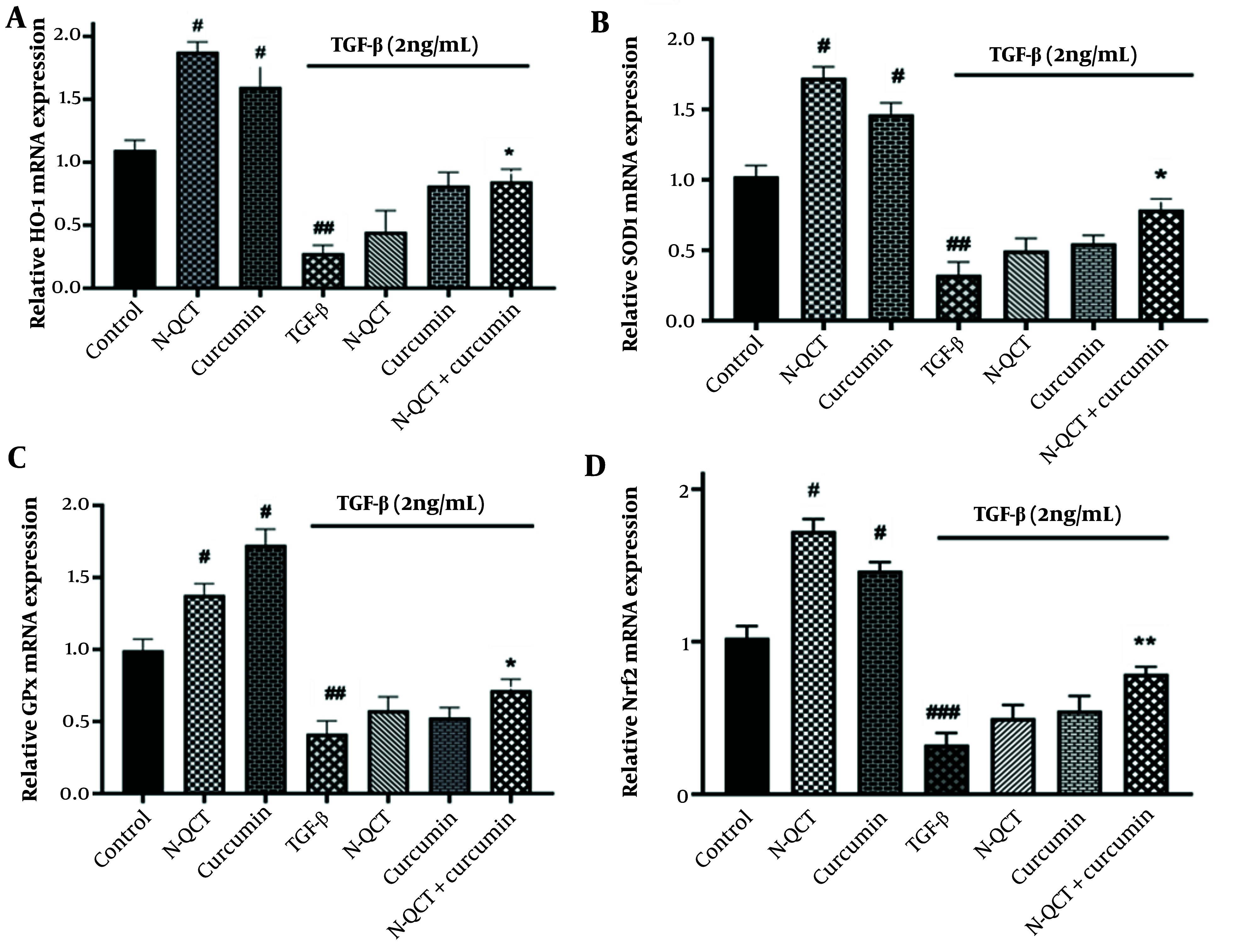
mRNA expression levels of antioxidant enzyme genes (A), (B), (C), and Nrf2 (D) in vascular smooth muscle cells (VSMCs), with and without TGF-β (2 ng/mL), following individual treatment with nano-quercetin (N-QCT), curcumin, or their combination. Statistical significance is indicated as follows: # P < 0.05, ## P < 0.01, ### P < 0.001 for comparisons between the TGF-β-stimulated group and control cells; and * P < 0.05, ** P < 0.01 for comparisons between other treatment groups and the TGF-β-treated cells (mean ± SD). The results were analyzed after three repetitions.

When comparing TGF-β-treated cells to the untreated group, there was a significant decrease in the transcription factor Nrf2 and the genes encoding antioxidant enzymes (SOD-1, GPx, and HO-1) (P < 0.0001). TGF-β-stimulated VSMCs treated individually with N-QCT and curcumin showed a slight increase in the expression of the Nrf2 transcription factor and the genes encoding antioxidant enzymes (SOD-1, GPx, and HO-1). Similarly, comparing the group treated with TGF-β alone (2 ng/mL) to the group treated with the combination of both drugs revealed a significant increase in Nrf2 mRNA expression (Figure 5D) and in the antioxidant enzymes (SOD-1, GPx, and HO-1) (Figure 5A - C).

### 4.9. Phosphorylation of Extracellular Signal-Regulated Kinase 1/2 and Nrf2 Proteins in TGF-β-Treated Vascular Smooth Muscle Cells with Curcumin and Nano-Quercetin

**Figure 6.**
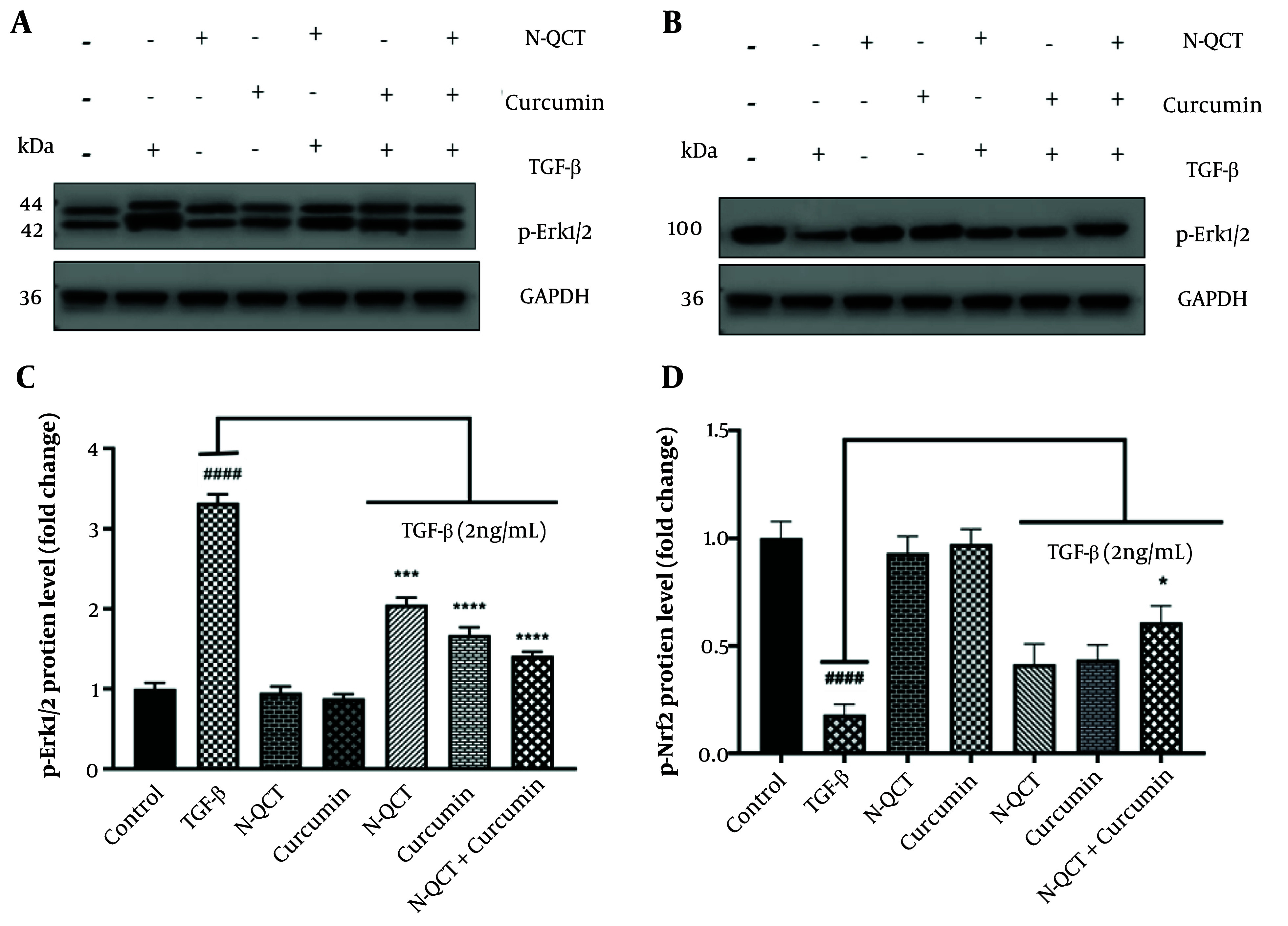
Effects on p-Erk1/2 and p-Nrf2 expression levels following treatment with nano-quercetin (N-QCT) and curcumin, either individually or in combination. Vascular smooth muscle cells (VSMCs) were treated for 24 hours with N-QCT (25.01 μM), curcumin (17.45 μM), or both drugs in combination. Protein expression levels of p-Erk1/2 (A) and p-Nrf2 (B) were assessed using Western blotting. (C) and (D) show the gray intensity analysis of the Western blot results for p-Erk1/2 and p-Nrf2, respectively. GAPDH was used as a loading control to ensure equal protein loading. Statistical significance is indicated as follows: #### P < 0.0001 for comparisons between the TGF-β-stimulated group and control cells; and * P < 0.05, *** P < 0.001, **** P < 0.0001 for comparisons between other treatment groups and the TGF-β-treated cells. Results are presented as mean ± SD and are based on three independent experiments.

Band density quantification was performed using ImageJ software, as outlined in Appendix 6 in Supplementary File. No differences were observed in the levels of p-Erk1/2 and p-Nrf2 proteins in untreated cells. Additionally, the protein levels of Erk1/2 and Nrf2 in unstimulated VSMCs were unaffected by either N-QCT or curcumin treatment. In TGF-β-stimulated VSMCs, a slight decrease in p-Erk1/2 protein expression and a slight increase in p-Nrf2 protein expression were observed in the groups exposed to N-QCT and curcumin compared to TGF-β-stimulated VSMCs (2 ng/mL) alone. In contrast, a significant increase in p-Erk1/2 protein expression (Figure 6A and C) and a significant decrease in p-Nrf2 protein expression (Figure 6B and D) were observed in cells treated with the combination of both TGF-β and the drugs.

## 5. Discussion 

Atherosclerosis is a chronic pathological condition characterized by oxidative damage and inflammation (30). This study reveals a TGF-β signaling mechanism involving both the Smad and non-Smad pathways (31). The Smad pathway enhances NOX/ROS expression and promotes vascular inflammatory responses, while non-Smad signaling activates the Erk pathway in human VSMCs. Reactive oxygen species induces oxidative stress, which influences cellular responses to both oxidative and electrophilic stresses (10). The nuclear factor erythroid 2-related factor 2 (Nrf2)-antioxidant response element (ARE) pathway regulates the expression of antioxidant enzymes in VSMCs (32). The ROS/Nrf2-ARE pathway facilitates the production and activation of these enzymes, thereby modulating cellular defense mechanisms against oxidative damage and maintaining cellular homeostasis (13, 15, 16).

No aggregate formation was detected in the drug release study, as shown in Figure 1B. The size distribution and uniformity of N-QCT particles, confirmed through TEM imaging, ensure stable and efficient drug release without aggregation. This is critical for maintaining consistent therapeutic efficacy over time. The study successfully integrated QCT into SLN using emulsification, enhancing its stability and bioavailability. This demonstrates the potential of SLNs as carriers for compounds with limited solubility. The efficient encapsulation and controlled release of QCT within these nanoparticles offer promising implications for advancing therapeutic approaches and refining drug delivery methodologies. The stability and uniform size distribution of QCT-loaded SLNs suggest enhanced drug delivery and improved therapeutic outcomes. The slow-release mechanism of N-QCT makes it a promising candidate for sustained drug delivery, improving treatment outcomes, reducing adverse effects, and enhancing patient adherence in atherosclerosis management. Furthermore, the combination of N-QCT and curcumin exhibits synergistic anti-inflammatory effects and modulates inflammation signaling pathways, enhancing the therapeutic potential of these compounds.

TGF-β plays a crucial role in atherosclerosis, with increased TGF-β1 expression observed in various models and clinical scenarios. This study investigates the impact of N-QCT and curcumin on inflammation in human VSMCs, focusing on their potential to modulate inflammatory responses and signaling pathways. Although curcumin's effectiveness is limited by poor water solubility, QCT can enhance its bioavailability by improving absorption (33).

The study found that both N-QCT and curcumin inhibited VSMC proliferation, with N-QCT exhibiting slightly higher cytotoxicity than curcumin. The discrepancies in IC_50_ values between N-QCT and curcumin in VSMC cells may be attributed to variations in the purity and quality of the QCT used in previous studies, highlighting the importance of controlled experimental conditions (34).

Additionally, the study examined the synergistic effect of N-QCT and curcumin on inhibiting VSMC cell growth. The results demonstrated a synergistic effect when the compounds were used together at their respective IC_50_ concentrations, suggesting that the combination of these compounds may have a more pronounced inhibitory effect on VSMC proliferation than each compound used individually (21, 35).

TGF-β, a critical factor in vascular diseases, is believed to exacerbate conditions such as restenosis and atherosclerosis by impairing VSMCs. Regulating ROS levels or enhancing antioxidant defenses could help mitigate TGF-β's adverse effects on VSMCs. The combination of curcumin and N-QCT effectively suppressed TGF-β-induced ROS production in VSMCs, suggesting a potential synergistic approach for treating vascular diseases. This aligns with existing research highlighting the anti-inflammatory and antioxidant properties of both compounds (36).

Findings from studies examining the antioxidant enzymes (SOD1, GPx, and HO-1) modulated by ROS in TGF-β-induced inflammatory responses provide valuable insights into the complex mechanisms underlying the pathogenesis of atherosclerosis (37). The N-QCT and curcumin, delivered through SLN, may reduce atherosclerosis-related inflammation by enhancing the expression of antioxidant enzymes. In response to ROS-induced inflammation, these compounds alter the Nrf2-ARE pathway and antioxidant enzymes, thereby influencing oxidative stress. Curcumin and N-QCT may increase antioxidant enzyme activity, reduce oxidative damage, and enhance cellular homeostasis by activating this pathway, supporting prior findings on oxidative stress and inflammation (38, 39). The study suggests that modulating Nrf2-ARE signaling pathways could lead to innovative therapeutic interventions for human diseases. Additionally, it explores the impact of combinatorial pharmacological agents on Smad and non-Smad signaling cascades, advancing our understanding of how signaling networks can be manipulated (40).

While individual treatments showed modest effects, the combination of N-QCT and curcumin resulted in significant reductions in oxidative stress and inflammation, as evidenced by the increased expression of p-Nrf2. This synergistic action suggests that both compounds interact with key signaling pathways, particularly through the activation of Nrf2, to enhance cellular defense mechanisms against oxidative stress. Figures 3 and 6 illustrate this enhanced effect, particularly through the modulation of the Nrf2-ARE pathway, leading to a more pronounced therapeutic outcome.

Atherosclerosis is a pathological condition characterized by oxidative stress and inflammatory responses within VSMCs, primarily triggered by the activation of NOX and the subsequent generation of ROS. A distinctive therapeutic strategy involves the synergistic interaction of N-QCT and curcumin, which attenuates the TGF-β/ROS/NOX signaling cascades (21, 35). This approach, aimed at addressing ROS-mediated inflammation and NOX activation, has the potential to impede the progression of atherosclerosis and reduce cardiovascular risk.

This study highlights the role of specific therapies in atherosclerosis by elucidating their effects on the TGF-β/ROS/Erk1/2 signaling pathway. Our findings revealed that N-QCT and curcumin, when combined, significantly mitigated TGF-β-induced Erk1/2 phosphorylation. Previous research by Zhang et al. and Yang et al. demonstrated that p38 MAPK and Erk are involved in Smad2L phosphorylation, influenced by curcumin's effects on CRP in VSMCs (41, 42). 

To explore the effects of N-QCT and curcumin on TGF-β-stimulated VSMCs, we performed western blot analysis to assess phosphorylated ERK1/2 and nuclear Nrf2 levels. The combination of N-QCT and curcumin yielded a more pronounced effect on TGF-β-stimulated VSMCs compared to either agent alone. Similarly, Shih et al. demonstrated that QCT affects the phosphorylation of Erk1/2, p-38, and JNK in rat aortic smooth muscle cells, which aligns with our findings (43).

Additionally, our results show that QCT and curcumin reduced total and phosphorylated Erk and AKT in MGC-803 cells, consistent with findings by Zhang et al. (44). Furthermore, Qin et al. demonstrated that curcumin inhibits growth and p-Erk1/2 activation in rat VSMCs. Our study confirms that N-QCT decreases total and phosphorylated Erk and AKT levels in MGC-803 cells, suggesting that N-QCT effectively regulates critical signaling pathways associated with cell proliferation and survival (45). 

### 5.1. Conclusions

The study reveals that combining QCT loaded into SLNs with curcumin effectively reduces inflammation and oxidative stress in TGF-β-stimulated VSMCs. The combination of these compounds led to a decrease in phosphorylated Erk 1/2 expression and an increase in nuclear Nrf2 activity, which enhanced antioxidant enzymes such as SOD1, GPx, and HO-1. This suggests that these natural compounds can target both canonical and non-canonical TGF-β/ROS/Erk1/2 signaling pathways, providing a comprehensive approach to combat atherosclerosis. The findings also support previous studies demonstrating the antioxidant and anti-inflammatory properties of QCT and curcumin, suggesting their potential to reduce the risk of cardiovascular disease.

ijpr-23-1-151428-s001.pdf

## Data Availability

The dataset presented in the study is available on request from the corresponding author during submission or after publication.
