# Supplementary material for: Dual Modulation of Canonical and Non-canonical TGF-β/ROS/Erk1/2 Pathways: Synergistic Activation of Nrf-2 and Antioxidant Enzymes (SOD1, GPx, HO-1) by Quercetin Loaded in Solid Lipid Nanoparticles and Curcumin in Atherosclerosis Therapy
Source: Iran J Pharm Res. 2024 Dec 13;23(1):e151428. doi: 10.5812/ijpr-151428 (PMC11892753; doi:10.5812/ijpr-151428)
Supplement: ijpr-23-1-151428-s001.pdf [file ijpr-23-1-151428-s001.pdf]

scatteroscope

*New Concept in Particle Size Analysis*

# Particle Size Analysis Report

01:37:41

## ¶ Data Information

- **Project Name** od
- **Data Name** od0.08-3
- **Sample Source**
- **Remarks**

## ¶ Measurement Conditions

| | | | |
|---|---|---|---|
| **Measurement Start Time** | 2009/01/01 01:34:41 | **Solvent** water | |
| **Measurement End Time** | 2009/01/01 01:34:44 | - **Refractive Index** | 1.3300 |
| **Exposure Type** | Single Exposure | - **Viscosity** | 0.9935 cP |
| **Total Exposure Time** | 1 sec | **Solute** water | |
| **Exposure Time Length** | 246 msec | - **Refractive Index** | 1.3300 |
| **Average Count Rate** | 29 kcps | - **Absorption Coefficient** | 0.0000 cm-1 |
| **Temperature** | 21 i/EC | **Machine Serial Number** | 2000-040 |

## ¶ Analysis Results

| | | | | | |
|---|---|---|---|---|---|
| **d(0)** | 39.3 nm / 63.8 nm | **d(5)** | 85.9 nm / 2.32 um | **# of Peaks** | 2 |
| **d(10)** | 97.3 nm / 2.71 um | **d(25)** | 123 nm / 3.43 um | 156 nm | 4.4 um |
| **d(50)** | 156 nm / 4.40 um | **d(75)** | 200 nm / 5.57 um | | |
| **d(90)** | 249 nm / 6.93 um | **d(95)** | 286 nm / 7.98 um | | |
| **d(100)** | 8.90 um / 17.4 um | | | | |

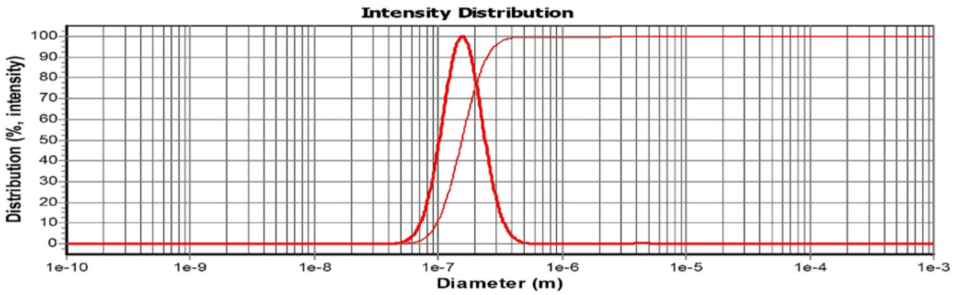


**Appendix 1**. The particle size distribution of quercetin and SLN was determined using dynamic light scattering (DLS)

**System**

| | | | |
|---|---|---|---|
| Temperature (°C): | 25.0 | Zeta Runs: | 30 |
| Count Rate (kcps): | 141.5 | Measurement Position (mm): | 2.00 |
| Cell Description: | Clear disposable zeta cell | Attenuator: | 6 |

**Results**

| | | | Mean (mV) | Area (%) | St Dev (mV) |
|---|---|---|---|---|---|
| Zeta Potential (mV): | -27.7 | Peak 1: | -26.7 | 100.0 | 7.86 |
| Zeta Deviation (mV): | 15.8 | Peak 2: | 0.00 | 0.0 | 0.00 |
| Conductivity (mS/cm): | 0.0147 | Peak 3: | 0.00 | 0.0 | 0.00 |
| Result quality : | **See result quality report** | | | | |

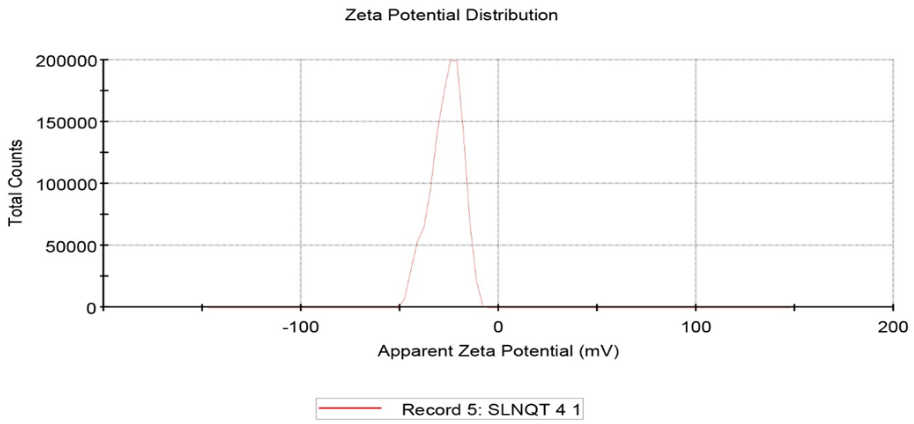


**Appendix 2.** The distribution of zeta potential in quercetin and solid lipid nanoparticles (SLN) was examined.

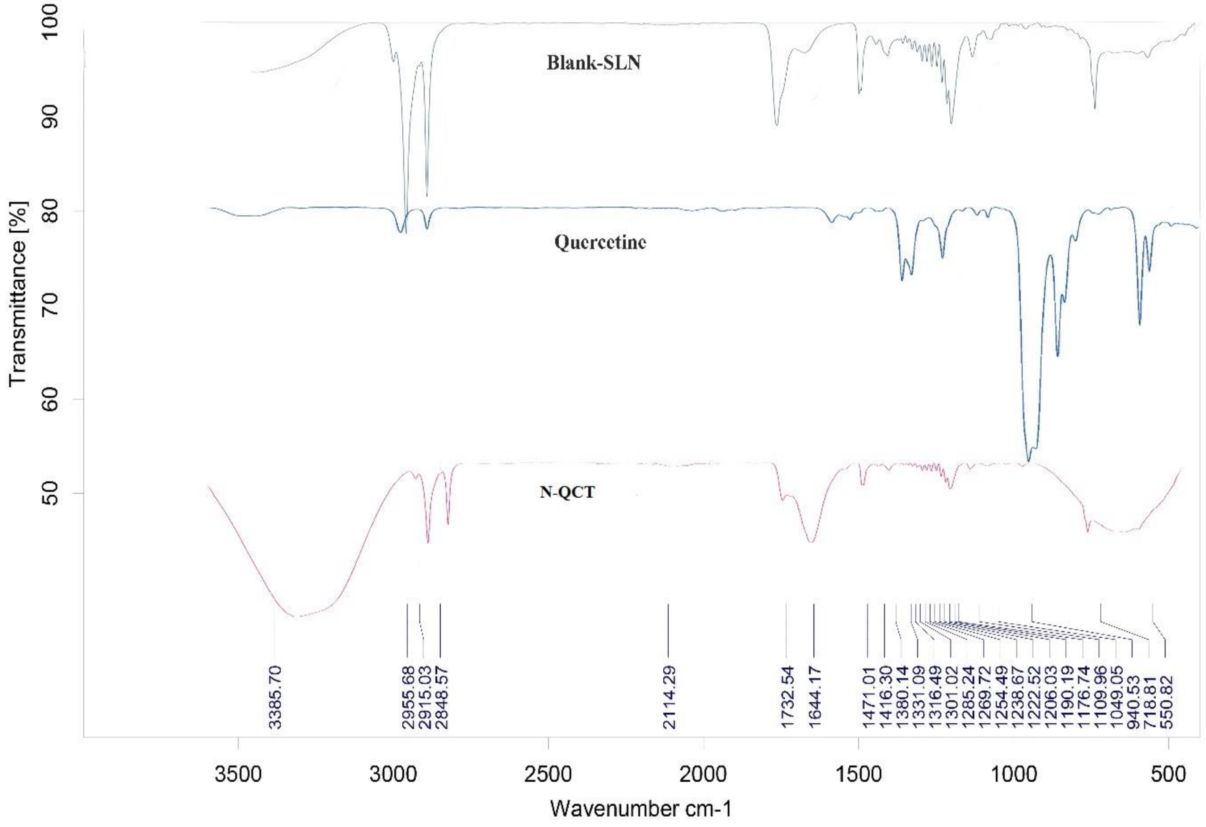


**Appendix 3**. QU, Blank-SLN, and N-QCT FTIR spectra

## Appendix 4. Formulation of QU-SLN.

| Sample(Formulatio) | Drug-Lipid Ratio | Copritol(mg) | Quercetin (mg) | Oleic Acid (gr) | PVA(1 %) (ml) | Lecitin (gr) | EE(%) | LD(%) |
|---|---|---|---|---|---|---|---|---|
| QU-SLN1 | 1:1 | 100 | 100 | 0.25 | 4 | 0. 5 | 65.6 ± 3.3 | 1.02% ± 2.6 |
| Blank-SLN1 | - | 100 | - | 0.25 | 4 | 0. 5 | | |
| QU-SLN2 | 1:5 | 500 | 100 | 0.25 | 4 | 0. 5 | 74.4 ± 2.8 | 1.24% ± 2.9 |
| Blank-SLN2 | - | 500 | - | 0.25 | 4 | 0. 5 | | |
| QU-SLN3 | 1:10 | 1000 | 100 | 0.25 | 4 | 0. 5 | 87.3 ± 3.8 | 1.62% ± 3.1 |
| Blank-SLN3 | - | 1000 | - | 0.25 | 4 | 0. 5 | | |
| QU-SLN4 | 1:30 | 3000 | 100 | 0.25 | 4 | 0. 5 | 96.6 ± 2.3 | 1.78% ± 3.3 |
| Blank-SLN4 | - | 300 | - | 0.25 | 4 | 0. 5 | | |
| QU-SLN5 | 1:47.5 | 4750 | 100 | 0.25 | 4 | 0. 5 | 99.3 ± 1.2 | 1.81% ± 2.1 |
| Blank-SLN5 | - | 4750 | - | 0.25 | 4 | 0. 5 | | |

SD: standad deviation, LD: loading agent, EE: encapsulation; (mean ± SD), The results have been analyzed after three repetitions.

## Appendix 5. The mole fraction of each combination of N-QCT and Curcumin

| Curcumin | 10 | 25 | 50 | 100 | 150 |
|---|---|---|---|---|---|
| N-QCT | | | | | |
| 10 | 78.44 | 67.89 | 77.21 | 59.07 | 46.54 |
| 25 | 66.97 | 62.89 | 50.67 | 44.62 | 31.07 |
| 50 | 57.19 | 41.27 | 54.78 | 28.09 | 42.19 |
| 100 | 43.27 | 33.57 | 39.07 | 18.97 | 39.41 |
| 150 | 22.08 | 28.07 | 18.08 | 13.69 | 17.97 |

## Appendix 6. Quantification of Western Blots with Image J

| Step | Description |
|---|---|
| 1. Scanning the Western Blot Film | The Western blot film was scanned at high resolution to ensure accurate detection of protein bands. |
| 2. Downloading the Image | The scanned image of the Western blot was saved in a compatible format (e.g., TIFF, JPEG) for further analysis |
| 3. Installing ImageJ | ImageJ software was installed on the computer. The version used was [specify version]. |
| 4. Setting the Measurement Criteria | Open the image file of the protein band scan in ImageJ. Select the "Rectangle" tool from the top toolbar to outline all protein bands of interest. |
| 5. Analyzing the Gel | Navigate to the “Analyze” menu on the top toolbar, select “Gels,” and then choose “Select First Lane” (Ctrl+1) for horizontal comparison of bands. Then, go to the “Analyze” menu, select “Gels,” and click on “Plot Lanes” to generate a plot of the intensity profiles across the lanes. |
| 6. Drawing and Measuring | Use the “Straight” tool from the top toolbar to draw across the plot at the base of the chart, helping to measure the intensity accurately. Select the “Wand” (tracing tool) from the top toolbar and click on each area of the plot to identify and highlight the peaks corresponding to the protein bands. |
| 7. Labeling and Exporting Results | Return to the “Analyze” menu, select “Gels,” and then click on “Label Peaks” to label the peaks on the plot. Click “OK” to finalize. Copy the results from the ImageJ output and paste them into Excel or Prism software for further analysis and visualization. |
